# Case report: nocardia infection associated with ectopic cushings

**DOI:** 10.1186/1472-6823-14-51

**Published:** 2014-06-20

**Authors:** Azra Rizwan, Aqiba Sarfaraz, Abdul Jabbar, Jaweed Akhter, Najmul Islam

**Affiliations:** 1 Section of Endocrinology, Department of Medicine, Aga Khan University Hospital, Stadium Road, P.O. Box 3500, Karachi 74800, Pakistan

**Keywords:** ACTH Adrenocorticotrophin Hormone, TS trimethoprim-sulphamethoxazole, IPSS inferior petrosal sinus sampling

## Abstract

**Background:**

Cushing’s syndrome results from exposure to excess glucocorticoids. Ectopic Cushings is endogenous ACTH dependant form of Cushing’s associated with markedly raised ACTH and cortisol levels. This leads to an impaired immune response, setting the stage for occurrence of opportunistic infections. Nocardiosis is a gram positive bacterial infection caused by aerobic actinomycetes in genus *Nocardia*. We report a series of patients diagnosed with ectopic Cushings, having pneumonia with *Nocardia* spp. In one of these cases, the manifestations of Cushing’s disappeared with treatment for *Nocardia*.

**Case presentation:**

Two middle aged men of Asian descent presented to the Endocrine clinic: the first with history of exertional shortness of breath, and weight loss for 1 year, the other with facial swelling, disturbed sleep and lethargy for a month. The third case was a young Asian male who presented with progressive weakness & weight loss for 2 months. All three patients had uncontrolled hypertension, high blood sugars & were hypokalemic (K: 2.52, 2.9, 1.5 mmol/l); 24 hour urine cortisol was elevated at 2000, 27216 and 9088 (32-243 ug/24 hours); ACTH 68.5, 159, 255 [0–48 pg/ml), respectively. Their MRI pituitary was normal, inferior petrosal sinus sampling revealed no central peripheral gradient. CT chest of these subjects demonstrated cavitatory lung lesions; microscopic analysis of respiratory samples was suggestive of infection with *Nocardia* spp. Histopathology of bronchoscopic-guided biopsy revealed no malignancy. Antihypertensives, insulin, potassium replacement, ketoconazole & trimethoprim-sulphamethoxazole (TS) were initiated. The patients’ symptomatology improved & cavitatory lesions resolved with treatment. The primary source for the ectopic cushings remained unknown. The first case required bilateral adrenalectomy. The second case followed a progressively downhill course leading to death. In the third case, we were able to completely taper off ketoconazole, potassium, insulin & antihypertensives, after starting TS.

**Conclusion:**

Opportunistic infections are known to be associated with Cushing’s syndrome, and higher levels of glucocorticoid secretion are found in patients with ectopically produced ACTH. Pulmonary nocardiosis is important differential to consider. This series includes the first case reported in which signs and symptoms of cushings subsided after treatment of *Nocardia.*

## Background

Cushing’s syndrome results from prolonged exposure to excess glucocorticoids. This could be due to an increased endogenous production of steroids, or could result from overzealous use of medication containing steroids [1]. Ectopic Cushings is an endogenous ACTH dependant form of Cushing’s that accounts for 10% of ACTH dependant causes [2]. This form of Cushing’s is associated with markedly raised ACTH, and, subsequently, cortisol levels. Elevated cortisol levels lead to an impaired immune response by neutrophils & macrophages and diminished recruitment of these inflammatory cells into the infected site. This sets the stage for the occurrence of bacterial and fungal opportunistic infections [2]. Nocardiosis is a gram positive bacterial infection caused by aerobic actinomycetes in the genus Nocardia [3].

We report a series of patients diagnosed with ectopic Cushings, with microscopic analysis suggestive of pulmonary nocardiosis. In one of these cases, the manifestation of Cushing’s disappeared simply with treatment for *Nocardia*.

## Case presentation

### Case 1

A 53 year gentleman, banker by profession, first presented to us in 2006, with exertional shortness of breath and lethargy for a period of 1 year. Associated with this was significant weight loss (6 kg over a 4 month period). His appetite had significantly decreased, although there had been no cough or fever. He had also been facing difficulty in climbing stairs since the previous month. Hypertension was diagnosed 6 months prior to presentation. There was no history of Tuberculosis or Type 2 diabetes. Addictions included a history of smoking 30–35 cigarettes/day for the past 25-30 years. There was no history of steroid intake, including that of traditional homeopathic or herbal medication consumed extensively in the Indo-Pak region.

On examination, his blood pressure was 160/90 mmHg. He had a pulse of 86 beats/minute, which was regular. His weight was 71 kg, height of 170 cm. He was mildly anaemic, had no goiter or clinically palpable nodule. There was no gynecomastia, no striae, no evidence of easy bruisability or pruritis. His visual fields were normal; there was no papilledema. There was significant proximal myopathy. Chest examination revealed crackles at left lung base. The rest of the systemic examination was normal.

**Figure 1:**
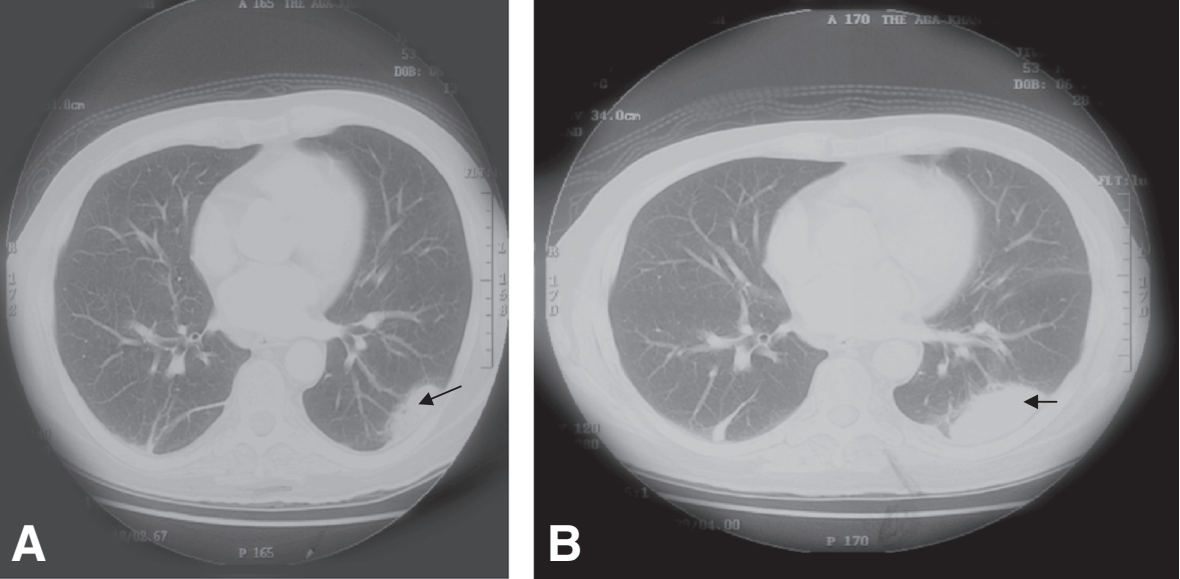
**CT Chest before treatment for Nocardia (Case 1). (A)** Cavitatory lesion (3.6×2.5×1.8 cm) in left lower lobe lung with adjacent alveolitis. **(B)** Soft tissue density mass left lower lobe (5.2×3.5×2.8 cm)-near doubling of lesion within span of 14 days.

He was severely hypokalemic (K: 2.52). Screening for Cushings revealed a 24hour urine cortisol of 2000 ug/24 hrs (32–243), an overnight dexamethasone suppression test of 31.7 ug/dl (<1.8 ug/dl), while baseline ACTH was 68.5 pg/ml (0–46). Post overnight 8 mg dexamethasone suppression test revealed a value of 18.9 ug/dl (baseline cortisol: 20 ug/dl).Chest radiography demonstrated left lower haziness, while CT chest with contrast revealed a cavitatory lesion (3.6×2.5×1.8 cm) in left lower lobe lung with adjacent alveolitis (Figure 1). There was no mediastinal and hilar lymphadenopathy.

**Figure 2:**
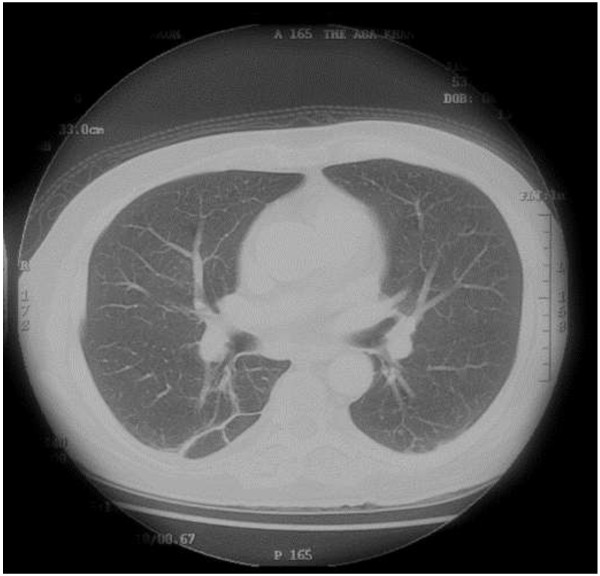
(Case 1) CT Chest after treatment for Nocardia (resolution of cavitatory lesion).

The history & laboratory work-up appeared to be consistent with ectopic Cushings, with the obvious source being the lungs. Hence, we proceeded with a CT guided biopsy of the lungs, which was repeated a week later. On both occasions, biopsy revealed chronic inflammatory changes, with no evidence of malignancy. Given an inconclusive biopsy result, and ACTH levels that were not too high, we requested for an MRI of the pituitary to look for a pituitary adenoma (Cushing’s disease). This revealed a 0.5×0.5 cm lesion, not showing typical post contrast signal of pituitary adenoma. A subsequent IPSS (inferior petrosal sinus sampling) and sampling from other sites revealed no gradient of ACTH level (Table 1). In the meantime, trimethoprim-sulphamethoxazole (TS), at a dose of 10 mg trimethoprim/kg/day (710 mg/day), had empirically been started for infection with *Nocardia* spp. by the pulmonologist keeping in mind a repeat CT Chest (Figure 1b) showing the patient’s lung lesion to have almost doubled within a span of 14 days. This is a sign more consistent with infection, since the average doubling time of lung cancers reported in most studies is at least 20 days [4]. At this stage, the pulmonologist felt that a bronchoscopy would not help in the case of such a deep seated peripheral lesion. A Cardiothoracic opinion deemed the patient’s overall condition too sick for a video assisted transthoracic surgery/open lung biopsy. His condition started improving slightly with conservative therapy which included potassium replacement, insulin, anti hypertensives, ketoconazole and TS, after which he was discharged on request. Two months later, he was readmitted with worsening shortness of breath, worsening proximal myopathy and bone pains. An X ray chest revealed left lower lobe collapse. A bronchoscopy was done. Gram stain and partial acid fast staining of bronchial washings was suggestive of infection with *Nocardia* spp. (Table 2). However, the culture did not grow *Nocardia* spp. possibly due to prior antibiotic treatment. The dose of TS was maximized to 15 mg/kg/day (1200 mg/day), and the medication was continued for a period of 6 months. Culture for *Mycobacterium tuberculosis* remained negative. The histopathology sample of the bronchoscopic guided biopsy was not consistent with malignancy. A repeat petrosal sinus sampling was done, again revealing no central-peripheral gradient (Table 1). Since the source of the ectopic cushings could not be identified and his general condition was worsening, he underwent bilateral adrenalectomy. Following the procedure, his condition slowly improved. A repeat CT chest showed resolution of the cavitatory lesion detected earlier (Figure 2).

**Table 1:** Inferior petrosal sinus sampling (Case 1)

**ACTH levels**	**16/07/2006**	**13/12/2006**
RT INF Petrosal 1	127	67.7
RT INF Petrosal 2	113	70.7
LT INF Petrosal 1	126	63.5
LT INF Petrosal 2	136	71.8
Peripheral 1		59.8
Peripheral 2		43.7
Peripheral 3	113	81.4
RT Atrium	134	
IVC	111	62.5
SVC	141	70.5
INFRA Renal		62.3
RT Internal Jugular		95.2
LT Internal Jugular		60.1

**Table 2:** Microbiology data details

	**Smear**	**Culture**	**Sensitivity [S-Sensitive, R-Resistant]**
**Case 1**	Suggestive of Nocardia	“Nocardia fails to grow on culture”	Not applicable
**Case 2**	Suggestive of Nocardia	Growth of Nocardia spp	Amoxicillin/clavulanate - S
			Ampicillin -R
			Amikacin-S
			Imipinem-S
			Gentamicin-R
			Ceftriaxone-S
			Cefotaxime-R
			Erythromycin-R
			Ofloxacin -R
			Co-trimoxazole-S
			Tetracycline-R
**Case 3**	Suggestive of Nocardia	Growth of Nocardia spp	Amoxicillin/Clavulanate-S
			Ampicillin-S
			Amikacin-S
			Imipinem-S
			Gentamicin-S
			Ceftriaxone-S
			Erythromycin- S
			Co-trimoxazole-S
			Tetracycline-S

### Case 2

A 54 years old businessman presented to the Endocrine clinic. He was hypertensive, non-smoker, but tobacco pan chewer. For 3 weeks prior to presentation he had been having swelling of the feet and face, disturbance in sleep pattern, and was irritable. He reported weight loss of 10 kg over the previous 45 days. Medication he had been prescribed were losartan, atenolol, spironolactone.

On examination, he was found to have blood pressure of 150/90 mmHg. He was having significant pedal edema, ecchymoses, lingual ulcers and central obesity. His right leg was disproportionately swollen, and red. Laboratory investigations revealed severe hypokalemia [K:2.9] and an elevated fasting blood sugar (FBS) of 200 mg/dl. An ultrasound dopplers revealed no deep venous thrombosis. He was admitted for work-up & further management for uncontrolled hyperglycemia, hypertension, hypokalemia and right leg extremity cellulitis. His antihypertensive medication [losartan, aldactone] and potassium supplementation were upgraded. Subcutaneous insulin was initiated for blood sugar control.

Further work-up revealed an elevated urine cortisol (27216) and overnight dexamethasone suppression test (134 ug/dl). A subsequent ACTH level was raised at 159 (0–48). MRI pituitary was normal. His x ray chest revealed a cavitatory lesion adjacent to left heart border, and a possible left pleural effusion. A subsequent CT chest with contrast confirmed a left lung cavitatory lesion. A CT guided biopsy of lung lesion was done, with the section revealing blood admixed with acute inflammatory cells, predominantly neutrophils. No tumour tissue was identified. The patient then had an episode of haemoptysis, prompting bronchoscopy. No endobronchial lesion was seen. Bronchial washings revealed no malignant cells, budding yeast cells or hyphae. Moderate branching gram positive filamentous rods were seen and the modified acid fast stain was positive, which was highly suggestive of *Nocardia*. Subsequent culture grew *Nocardia* spp. (Table 2) Acid fast bacilli were not seen. A repeat CT scan a week later revealed development of new cavitatory lesions in the left lung upper and lower lobe.

He was started on trimethoprim-sulphamethoxazole (TS) at 15 mg trimethoprim/kg (1200 mg/day) for the *nocardia*, and discharged in a stable condition, with advice to continue TS for 6 months. A follow-up CT chest 3 months later revealed complete resolution of cavitatory lesions previously seen in upper & lower lobe left lung. The patient then got lost to follow-up.

A year later, he presented with worsening bone pains, for which a whole body skeletal scintigraphy was requested. The latter showed widespread bony metastasis. CT chest and abdomen with contrast revealed multiple tiny nodules in both lungs and enlarged lymph nodes within the mediastinum. Multiple well-defined low attenuated lesions were noted in the liver, while 3 peripherally placed lesions identified in spleen. Both adrenals were bulky. Numerous enlarged paraaortic and peripancreatic lymph nodes were identified. A CT guided biopsy of a paraaortic lymph node detected this time showed a metastatic adenocarcinoma.

Subsequently, he was readmitted with worsening shortness of breath. Chest radiography showed hilar vascular congestion with pulmonary vascular congestion in lower lung zones bilaterally. He was started on broad spectrum intravenous antibiotics, and was given intravenous fluids judiciously. On the second day of admission, he became unresponsive, pulseless and hypotensive. Since the code had been decided as no shock and no CPR, he was not intubated, and death was declared shortly.

### Case 3

A 38 year male, hypertensive for 5 years, and an ex-smoker was admitted with chief complaints of progressive weakness, particularly on climbing stairs and standing from squatting position, and with excessive weight loss for 2 months. He had also been diagnosed with type 2 diabetes a month prior to presentation. On physical examination, he was afebrile with a blood pressure of 160/100 mmHg and respiratory rate of 22 breaths per minute. He had ecchymoses on his arms and legs. Respiratory examination revealed diminished breath sounds at the lung bases with some crackles. On neurologic examination, he was well oriented to time, place and person. Power was 3/5 on both lower limbs with decreased reflexes. Initial laboratory results showed severe hypokalemia (K 1.5 mEq/L), Random blood sugar (RBS) of 400 mg/dl, TSH: 5.68, Serum Renin 0.51 ng/ml (supine 0.15-2.33 ng/ml/hr, standing0.31-3.95 ng/ml/hr), Aldosterone 15 ng/dl (standing.4-31 ng/dl recumbent 1-16 ng/dl). His ACTH was 255 pg/ml, 24 hour urine cortisol 9088 ug/24 hours. Serum cortisol was 192 ug/dl, which failed to suppress with high dose (8 mg) dexamethasone, remaining at 160 ug/dl.

**Figure 3:**
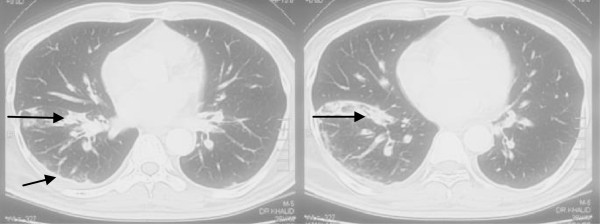
(Case 3) CT Chest before treatment for Nocardia (Consolidation right anterobasal segment & pleural effusion).

**Figure 4:**
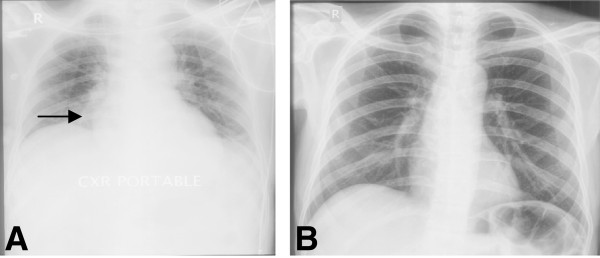
**Chest X-ray before and after treatment for Nocardia (Case 3). (A)** Pre-treatment consolidation right anterobasal segment & pleural effusion. **(B)** Post-treatment resolution of right anterobasal segment consolidation and pleural effusion.

Findings on clinical examination and laboratory evaluation raised the suspicion of ectopic ACTH secretion originating from lungs. Chest imaging showed segmental consolidation/collapse of right anterobasal segment and bilateral pleural effusion (Figures 3 and 4). Subsequent bronchoscopy identified no lung mass but bronchoalveolar c/s grew *Nocardia* spp*.* (Table 2). MRI pituitary and CT abdomen showed no abnormality. Inferior petrosal sinus sampling showed no central-peripheral gradient (Table 3).

He was started on TS at 15 mg trimethoprim/kg/day-930 mg/day. Ketoconazole, potassium replacement, insulin and antihypertensive medication (aldactone and angiotensin receptor blocker) were optimized. On this regimen, the patient’s condition improved and he was discharged home. On follow up visits, the doses of his medication were tapered down as his symptoms and biochemical profile improved. Follow-up chest x-ray was normal (Figure 4b). [Follow up CT scan was not done due to financial limitations]. To date (from 2011 to 2014), he has been off ketoconazole. His serum potassium and 24hour urine cortisol have remained within the normal range. His blood sugars are well- controlled off insulin therapy.

**Table 3:** Inferior petrosal sinus sampling (Case 3)

**ACTH Levels**	**3/11/2010**
RT INF Petrosal 1	374
RT INF Petrosal 2	356
LT INF Petrosal 1	376
LT INF Petrosal 2	371
SVC	358
IVC	326
RT Peripheral	356

### Microbiological methods

Nocardia was identified using standard phenotypic methods [5]. On gram stain from specimen, (Sputum, tracheal aspirates, Bronchoalveolar lavage), Nocardia was suspected when thin beaded Gram positive branching rods were seen. A modified partial acid fast stain was then performed. If these branching rods were partial acid fast positive, a preliminary report was issued stating “Gram positive branching rods that are partial acid fast positive suggestive of infection with *Nocardia* spp. are seen”. Recommended quality control strains were used for Gram staining and partial acid fast staining. Growth of *Nocardia* spp was identified on colony morphology, musty odor, gram stain morphology, partial acid fastness, presence of aerial hyphae, decomposition of casein, tyrosine, xanthine, hypoxanthine, urease production and gelatin hydrolysis. Antimicrobial susceptibility testing was performed using Kirby Bauer disc diffusion method [6]. S. *aureus* ATCC 29213, P. *aeruginosa* ATCC 27853 and E.*coli* ATCC 35218 (for amoxicillin-clavulanic acid) were used for quality control of susceptibility testing.

## Conclusions

Our case series illustrates the different ways in which Nocardiosis can present in the setting of Ectopic Cushings. Ectopic Cushings is a well recognized cause of Cushing’s syndrome, usually characterized by remarkably elevated levels of ACTH, elevated 24hour urine cortisol levels and severe/resistant hypokalemia, which can manifest as proximal myopathy [1,2]. All 3 subjects had uncontrolled hypertension and high blood sugars. They were hypokalemic (K: 2.52, 2.9, 1.5 mmol/l); 24hour urine cortisol was elevated at 2000, 27216 and 9088 (32-243 ug/24 hours); ACTH 68.5, 159, 255 [0–48 pg/ml], respectively. Their MRI pituitary showed no adenoma, and inferior petrosal sinus sampling had revealed no central peripheral gradient.

In the first case, *Nocardia* spp produced a rapidly expanding cavitatory lesion in the left lung, and later left lower lobe collapse. The lung lesions eventually resolved with institution of appropriate antibiotic-trimethoprim-sulphamethoxazole (TS). *Nocardia* failed to grow on culture. This could have been a consequence of previous antibiotic use. In the second case, left upper and lower lung cavitatory lesions were identified, with bronchial washings revealing growth of *nocardia* spp. Although appropriate coverage for *Nocardia* spp. with TS was given, followed by resolution of the lung lesions, the patient had underlying metastatic adenocarcinoma of unknown primary. This ultimately led to a rapid deterioration of his condition, and death. In the third case, CT chest revealed consolidation of right lung lobe and bilateral pleural effusion. Subsequent bronchoalveolar c/s grew *Nocardia* spp. In this patient, the patient’s physical condition and biochemical profile, including the potassium level, 24 hour urine cortisol & blood sugar, as well as pulmonary lesions, on follow-up chest X-ray, had completely normalized with the course of TS for *nocardia* spp. We were able to taper off his ketoconazole, potassium supplementation and insulin.

The remarkably elevated levels of ACTH usually encountered in ectopic Cushing’s lead to high levels of glucocorticoid secretion. Glucocorticoids cause immunosupression by inhibiting the phagocytic function of alveolar macrophages and neutrophils, thereby reducing mobilization of inflammatory cells into infected areas. This results in an increased incidence of opportunistic bacterial and fungal infections [7]. Our case series highlights the importance of considering *Nocardia* spp as a causative agent for the pulmonic manifestations of patients diagnosed with Cushing’s syndrome, particularly in the Ectopic Cushing’s subset.

Opportunistic infections in Cushings syndrome carry a high mortality and morbidity [2,8]. A variety of opportunistic infections have been demonstrated in the presence of endogenous cortisol production. Most popular are *Pneumocystis jirovecci*, *Cryptococcus neoformans* and *Nocardia* spp. [9]. Huang TP et al. report a case of a young man who developed fever and dyspnea, with progression of his lung lesions, during the course of his hospital stay for work-up of Cushing’s syndrome. Infection with *Nocardia* spp was found. Despite institution of appropriate antimicrobial therapy, the patient expired [8]. Graham BS et al. studied a series of patients with endogenous Cushings syndrome and opportunistic infections. They indicated that hypercortisolism may lead to masking of symptoms and signs of infection, at times leading to a delay in the diagnosis, which contributes to the high mortality rate [10]. Sutton BJ et al. report a case of Cushing’s syndrome and nocardiosis associated with a pulmonary carcinoid tumour, diagnosed on fine needle aspiration of a left lung nodule. Prior to treatment for the tumour, new nodules developed in the lungs, fine needle aspiration of which revealed the nodules to be infectious in nature. Gram staining demonstrated gram positive branching organisms, and specimen culture grew *Nocardia* spp. [3]. Their case was amongst the first reporting use of the fine needle aspiration technique to diagnose both the pulmonary tumour, as well as the associated infectious complication arising from the hypercortisolism resulting from the tumour [3]. Boscaro M et al. report a patient with a disseminated form of nocardiosis in the setting of hypercortisolism resulting from bilateral nodular hyperplasia [11]. In a published series, ectopic production of ACTH has been responsible for the majority of opportunistic infections related to Cushing syndrome. This is most likely because the highest levels of glucocorticoid secretion are found in patients with ectopically produced ACTH [8,9]. Hoshino T et al. report a case of a young woman who presented with dyspnea. Chest X-ray examination showed an infiltrative shadow with pleural effusion. Pleural fluid obtained by thoracentesis showed *Nocardia* spp. Subsequently, she was diagnosed as a case of Cushings syndrome on the basis of low dose dexamethasone suppression test [12]. Sudou A et al. also report a case of pulmonary *Nocardia otitidis-caviarum* infection in a 35-year-old man with Cushing's disease. Chest radiograph showed a large bilateral mass in the lung fields. Sputum culture revealed *Nocardia otitidis-caviarum*[13]. Despite having received appropriate antibiotics, the patient expired due to respiratory failure. Tanaka M et al. also report a case of nocardia causing cerebral abscess in a patient with Cushing’s syndrome. The patient was admitted with complaints of general fatigue, thirst and backache. A diagnosis of Cushing's syndrome was made on the basis of elevated serum levels of cortisol and adrenocorticotropic hormone (ACTH). Pituitary imaging was completely normal. During hospital stay, he developed hemiplegia. Cerebral abscess in the right frontal lobe was demonstrated by brain CT scan. Surgical drainage was performed and *Nocardia* spp was isolated from the drained pus [14].

Other opportunistic infections such as cryptococcosis, aspergillosis, pneumocystosis and risk of pulmonary involvement in the setting of herpes viral infections, such as Cytomegalovirus and Varicella zoster virus, also need to be considered in a patient with Ectopic Cushing’s [8].

To the best of our knowledge, no case to date has been reported in the literature in which treatment of the *Nocardia* spp. resulted in disappearance of the clinical, biochemical and radiologic manifestations of the Ectopic Cushing’s (as in case 3). This included the severe proximal myopathy, refractory hypokalemia, hypercortisolism and the pulmonary lesions that the patient had presented with. We were able to withdraw all therapy (including ketoconazole). The patient has remained symptom-free till date (years 2011 to 2014). This suggests that nocardiosis may not only result from hypercortisolism, but can also itself lead to raised cortisol levels, and the manifestations of Cushing’s syndrome. The mechanism by which *Nocardia spp.* can cause Cushing’s syndrome is not clear, and, therefore, needs to be investigated further.

Opportunistic infections are known to be associated with hypercortisolism, and higher levels of glucocorticoid secretion are found in patients with ectopically produced ACTH. Pulmonary nocardiosis is an important differential to consider. This series includes the first case reported in which the signs and symptoms of Cushing’s subsided simply after antibiotic treatment of Nocardia.

### Consent

Written informed consent was obtained from all three patients for publication of this case report. Copies of the written consent statements are available for review by the Series Editor of this journal.

## Abbreviations

ACTH: Adrenocorticotrophic hormone; TS: Trimethoprim-sSulphamethoxole; IPSS: inferior petrosal sinus sampling; AFBC/S: Acid fast bacillus culture & sensitivity; FBS: Fasting blood sugar; RBS: Random blood sugar; TSH: Thyroid stimulating hormone; Nocardia spp: Nocardia species; CPR: Cardiopulmonary resuscitation.

## Competing interests

The authors declare that they have no competing interests.

## Authors’ contributions

AR and AS led the conception and design, acquisition of data, review of literature, and drafted the manuscript. NI, AJ and JA reviewed the manuscript and gave the concept of research paper, and critically reviewed the manuscript. All authors read and approved the manuscript.

## Authors’ information

AR is Fellow of the College of Physicians & Surgeons of Pakistan. She is Consultant in Endocrinology, Diabetes & Metabolism, Department of Medicine and Masters in Clinical Research, Aga Khan University Hospital. She was involved in the medical management of the first two patients. AS is Fellow of the College of Physicians & Surgeons of Pakistan. She is Fellow in Endocrinology, Diabetes & Metabolism, Department of Medicine, Aga Khan University Hospital. She was involved in the medical management of the third patient. AJ is Fellow of the Royal College of Physicians of London. He is Professor & Consultant Endocrinologist, Department of Medicine, Aga Khan University Hospital. He was the second patient’s primary physician & endocrinologist. JA is Fellow of the Royal College of Physicians of London. He is Professor & Consultant Endocrinologist, Department of Medicine, Aga Khan University Hospital. He was the third patient’s primary physician & endocrinologist. NI is Fellow of the Royal College of Physicians of London. He is Professor & Consultant Endocrinologist, Department of Medicine, Aga Khan University Hospital. He is also the Director Endocrinology, Diabetes and Metabolism Fellowship Program, Aga Khan University Hospital. He was the first patient’s primary physician & endocrinologist.

## Pre-publication history

The pre-publication history for this paper can be accessed here:

http://www.biomedcentral.com/1472-6823/14/51/prepub
